# From the Body Image to the Body Schema, From the Proximal to the Distal: Embodied Musical Activity Toward Learning Instrumental Musical Skills

**DOI:** 10.3389/fpsyg.2020.00101

**Published:** 2020-01-31

**Authors:** Jin Hyun Kim

**Affiliations:** Department of Musicology and Media Studies, Humboldt University of Berlin, Berlin, Germany

**Keywords:** action/passion, corporeality, body image, body schema, embodiment, existential feelings, instrumental musical skills, proximal/distal

## Abstract

A recent paradigm shift in music research has allowed scholars to examine the macro- and micro-processes taking place within musical performance and underlying cognitive processes. Tying in with phenomenological theories of embodied perception and cognition, this paper focuses on bodily musical activity relevant to the acquisition of instrumental musical skills – the process of learning music. Dynamic interaction with musical instruments, accompanied by the interplay of action and passion, involves body image and body schema, whose status oscillates in different phases of the acquisition of instrumental musical skills; this interaction allows humans to direct attention from their bodily states – the proximal – to the quality of musical sounds and a unity of musical experience – the distal. It is thus argued that shaping music by means of playing a musical instrument can be conceived of as an embodied process, of understanding the forms of one’s own experience as related to the musical world that is created by one’s bodily activity.

## Introduction

Most acts of playing a musical instrument involve bodily activity based on diverse instrumental techniques; these in turn depend on the affordances and constraints that the musical instrument provides (Reybrouck, 2012; Altavilla et al., 2013; Krueger, 2014), and which the player perceives as such. Efforts to produce sounds effectively and refine one’s control of sound qualities (e.g., timbre) are a basis for the acquisition of instrumental musical skills – that can involve highly sophisticated bodily activity – even in the case of a digital instrument, enhanced with gestural interfaces (Ryan, 1992). Moreover, bodily activity based on instrumental musical skills which serve to generate and control sound can be characterized as “musical,” in the sense that it has an affinity to the characteristics of the perceptible musical sounds (e.g., duration, contour, and tension/relaxation) that it generates and shapes.

The acquisition of instrumental musical skills is not only relevant to professional musicians, but to every human being who strives for bodily activity dealing with sound in those extra-ordinary contexts (Dissanayake, 1992) where, unlike interaction in language, the activity is not necessarily directed toward a clear goal to be achieved (Cross, 2014). The acquisition of instrumental musical skills is therefore a subject both for the pedagogy of instrumental music and for theoretical and empirical music research investigating the processes taking place in music-making and musical listening.

This paper focuses on the extent to which the bodily activity involved in the acquisition of instrumental skills plays a role in understanding the forms of one’s own experience that are supposed to have a structural analogy to the characteristics of musical sounds that are generated and controlled by one’s bodily activity (see section “Understanding the Forms of Our Own Experiences”). Recent embodied approaches to cognition have led many scholars to re-think the concepts of cognition as well as the body – the latter regarding the relation between mind and body, and between body and environment (Gallagher, 2005; Thompson, 2007; Calvo and Gomila, 2008). Tying in with phenomenological theories of embodied perception and cognition, this paper attempts to characterize the physical activity involved in learning music within the context of the acquisition of instrumental skills as embodied musical activity.

## Discourses on Corporeality and Embodiment

The physical activity required to play a musical instrument is largely refined bodily motor activity – not including the programming activities used with the computer-as-musical-instrument (Mathews, 1963) that have recently given rise to and established a subgenre of computer music called “live coding” (Collins et al., 2003). What are we doing when we begin to learn how to play a musical instrument? We attempt to coordinate appropriate physical energy, corporeal postures and gestures to generate and control sounds. We often confront physical difficulties in playing the musical instrument, and develop the techniques used to overcome them, such as respiration and fingering techniques. Mastering such basic instrument techniques is, in general, the first step in the acquisition of instrumental musical skills. In this stage, the body is often treated as a machine, closely related to posthumanist visions of “a regulated control, technical transformation, or even elimination of the body that is experienced as something defective” (Becker, 2000, p. 22).

But the question of whether a conceptualization of the body as a thing is appropriate deserves thorough discussion. In discourses on corporeality carried out in the context of cultural and media studies in tandem with growing scientific and artistic interest in corporeal dimensions for some considerable time, the body is regarded – in line with Western modern thought – either as a thing or as a construct (List and Fiala, 1997; Barkhaus and Fleig, 2002). These discourses thus continue to amplify Cartesian mind-body dualism, according to which an incorporeal entity – *res cogitans* – is conceived of as clearly distinguishable from a corporeal entity – *res extensa*. When treated as a thing, the body is changeable and extendable; for example, fitness discourse revolves around the body’s physical performance, including its capacity, readiness and improvement, and therefore centers its mechanical processes. When treated as a construct, the body is considered a form of appearance accessible to the senses, as well as representing and making perceptible something transcending the body. The presence of the body as a construct serves as a representation of something incorporeal, and at the same time as a transparent means of making the essential substance – the being beyond the body (cf. Krämer, 2000) – accessible to the senses.

Likewise, it is remarkable that the traditional concept of “embodiment” as semantically related to “incarnation” (Di Stefano, 2019) – embodiment being an incarnation of a non-physical substance or the instantiation of an abstract substance that is supposed to be *beyond* and *prior to* the body – is largely in line with Cartesian mind-body dualism (Seifert and Kim, 2012). In this semantic variant, “embodiment,” characterized as a dualist concept of embodiment in the sense of “embodiment of” (Seifert and Kim, 2012, p. 84), refers to both the processuality that realizing, materializing and concretizing a non-bodily substance implies through the *nomen actionis*, and the result thereof. The processuality of an action is only related to corporeal processes that obtain a secondary and subsequent status. In other words: a *two-world ontology* is presupposed, incorporating a transcendental world that can be made available subsequently through its appearance and a world of experience that makes the former appear, and therefore makes it available (cf. Krämer, 2000). For instance, several research programs in cognitive robotics pursue an embodied approach in the sense that the studies of human abstract cognition involve corporeal processes concretely realizing cognitive capacities. A concrete example provided by the cognitive scientists and roboticists Rolf Pfeifer and Christian Scheier is the designing of “a module for wall-following within the agent” to achieve wall-following behavior, as opposed to designing “basic processes that together, interacting with the environment, engender this desired behavior” (Pfeifer and Scheier, 1999, p. 307). This means that research programs within cognitive science using a robot could be based on the notion of the “sense-think-act-cycle” (Clark, 2016, p. 176), and thus fail to scrutinize an epistemological and ontological dualism.

That being said, a radical transformation of thinking about the relation of the mind and the body has already begun, allowing for the establishment of a non-dualist concept of “embodiment” that can be characterized as “being embodied” instead of “embodiment of” (Seifert and Kim, 2012, p. 85). The embodied mind is regarded as a starting state, rather than a final state resulting from a process embodying an immaterial substance. More precisely, a state once conceived of as the result of a process – and that process having been understood as goal-directed – is considered to constitute a unity (cf. Seifert and Kim, 2012, p. 85). This is related to the concept of entelechy developed in Aristotle’s philosophy. Actuality (Greek: 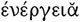, energeia; Latin: actus/actualitas), the process of achieving a goal, is distinguished from the potentiality (Greek: 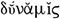, dunamis; Latin: potentia/possibilitas) of achieving a goal. Aristotle uses the term “entelechy” (Greek: 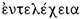, entelecheia; Latin: actualitas) to refer to actualization in general, the actuality resulting from actualization, or perfection of something in particular, as opposed to its mere potentiality; there is no separation between the goal (Greek: 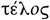, telos) to be achieved, the achieved goal or function (Greek: 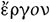, ergon), and the process of achieving the goal (Greek: 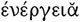, energeia). As such, the function to be achieved (e.g., percept; Greek: 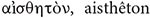), the function achieved (e.g., perception as the mental function; Greek: 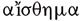, aisthêma) and the process of achieving the goal (e.g., the act or process of perception; Greek: 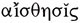, esthesis) become unified (cf. Seifert and Kim, 2007, p. 933f.). A non-dualist concept of “embodiment” can be best understood in this sense of entelechy.

The most important contributions to a new conceptualization of embodiment emerged from third-generation approaches in cognitive science after cognitivist and connectionist ones: “embodied cognition” (Clark, 1997, 2016), “embodied cognitive science” (Pfeifer and Scheier, 1999; Pfeifer and Bongard, 2007), “situated cognition” (Clancey, 1997; Robbins and Aydede, 2009), “interactionism” (Agre and Rosenschein, 1996; Agre, 1997), “interactivism” (Bickhard and Terveen, 1995), and enactivism (Varela et al., 1991; Hutto and Myin, 2013) among them (see for an overview Calvo and Gomila, 2008). They depart from the cognitivist/connectionist presupposition of a two-world ontology incorporating “a pregiven outer world” to be recovered and “a pregiven inner world” to be projected (Varela et al., 1991, p. 172) and focus on (1) the coupling of action and perception, i.e., motor and sensory processes and (2) the coupling of agent and environment. In other words, sensorimotor interactions serve as a basis for embodied cognition. Furthermore, embodiment necessarily implies situatedness. This means that a picture of embodied activity is dependent on the situation in which that activity occurs – in the case of embodied cognition, “relatively local” (Robbins and Aydede, 2009, p. 3). Therefore, cognition is not decoupled from its environment, and thus from the environment’s affordances or constraints. Approaches to embodiment and situatedness in cognitive science, especially in artificial intelligence, are necessarily closely connected with robotics, in which not only the physical body itself, but also the embodied and situated interaction of an agent with its environment – including other agents within it – is foregrounded (Pfeifer and Scheier, 1999; Pfeifer and Bongard, 2007). In this context, “embodiment” is defined by the roboticists Dautenhahn et al. (2002) as founded on the functional relation between the system and its environment, rather than as a feature of a cognitive system situated in an environment (Dautenhahn et al., 2002, p. 400).

## Bodily Activity in Phenomenological Theories of Embodied Perception and Cognition

Maurice Merleau-Ponty’s phenomenology of perception is one of the main reference points in contemporary discourse on embodiment, used in cognitive science as a philosophical framework to tackle the issue of embodiment. In contrast to intellectualism and empiricism, Merleau-Ponty’s phenomenology insists that “[w]e must rediscover, as anterior to the ideas of subject and object, the fact of my subjectivity and the nascent object, that primordial layer at which both things and ideas come into being” (Merleau-Ponty, 1962 [1945], p. 219). Going beyond the separation of subject and object, he discusses the distinctions between inside and outside, between consciousness and nature, and between mind and body.

Merleau-Ponty’s *La structure du comportement* (1942) and *Phénoménologie de la perception* (1945) examine the natures of behavior and perception in the relationship between the organism and its milieu, which are based on circular, not linear, causality (cf. Merleau-Ponty, 1963 [1942], p. 15). John Wild’s preface to the English edition of *La structure du comportement* (1942) summarizes Merleau-Ponty’s point that human behavior is “neither a series of blind reactions to external “stimuli,” nor the projection of acts which are motivated by the pure ideas of a disembodied, worldless mind. It is neither exclusively subjective nor exclusively objective, but a dialectical interchange between man and the world, which cannot be adequately expressed in traditional causal terms” (Wild, 1963, p. xiv). For Merleau-Ponty, there is no transcendental ego that is independent of the dimension of the acting body in space and time (cf. Merleau-Ponty, 1963 [1942]). Accordingly, he understands perception as a mode of access to the world. Likewise, his investigation of the structures of perception is based on a theory of the body, with which he wants to do away with the ideas of a separation between *res extensa* and *res cogitans*, as well as of inside and outside. Where classical psychology and physiology treats the body purely as an object that can be observed, Merleau-Ponty distinguishes the body proper (“le corps propre”) as a source of the entire world in which it is anchored, which cannot be observed from an external position (cf. Wild, 1963, p. xv).

Here, the site of perception is the body, considered to be an empirical ego, and it cannot be described with the matrix of “subjective” vs. “objective”: “I am my body, at least wholly to the extent that I possess experience, and yet at the same time my body is as it were a “natural” subject, a provisional sketch of my total being. Thus experience of one’s own body runs counter to the reflective procedure which detaches subject and object from each other, and which gives us only the thought about the body, or the body as an idea, and not the experience of the body or the body in reality” (Merleau-Ponty, 1962 [1945], p. 198f.). The relation between the body and physical objects is not causal; it can rather be described as an “intentional arc” (Merleau-Ponty, 1962 [1945], p. 157) within which the body and its milieu are interdependent. The body is therefore a decisive moment in the constitution of an objective world. Events exist only within the milieu of physical behavior and perceptual situations. There are no “blind reactions” (Wild, 1963, p. xiv) to physical facts that are outside an organism.

Merleau-Ponty’s theory grounds the stability of perception not in the capacity of consciousness, but in physical habitual actions – such as playing musical instruments – which precede the emergence of cognition. These repeated bodily actions – which he calls *habitus*, and which can be learned on the basis of imitation – are not merely mechanical processes, but forms of knowledge. The body functions as a means of communication with the world, exemplified by the relation between touching and being touched. Merleau-Ponty calls this “double sensations,” by which he means that, when one hand touches another, the transition from one function to another takes place; the hand that is being touched can also be a hand that touches (cf. Merleau-Ponty, 1962 [1945], p. 118f.). The relation between the act of perception and the perceived object, i.e., inside and outside, is captured as an inseparable, chiastic relation: “The world is wholly inside and I am wholly outside myself” (Merleau-Ponty, 1962 [1945], p. 401).

The German media theorist Barbara Becker highlights this kind of dynamic interaction with the world while investigating the concept of a body that serves neither as thing nor construct, but is considered to be embodied and situated within the world and entering into a dynamic interaction with the world. Taking up the phenomenology-minded philosophical tradition, Becker characterizes this interaction as the chiasmus of *action and passion* (Becker, 2000): “We touch and are touched: we act, and all the while our body responds to the silent offers coming from the respective environments we’re in, and from the world around us. And this counterpart is also always acting and reacting in us” (Becker, 2000, p. 44).

## Chiasmus of Action and Passion Related to a Dynamic Interaction With the Musical Instrument

What role does this chiasmus of action and passion play in the embodied activity involved in the acquisition of instrumental musical skills? When we encounter a new musical instrument, whether mechanical or digital, its physical or virtual materiality offers resistance; this allows us to engage in a dynamic interaction with the musical instrument, during which we act and react in relation to its materiality. Typically, we begin this interaction in the role of a “patient” – i.e., one being affected by an agent, to apply the anthropologist Alfred Gell’s term (Gell, 1998). During the time we are learning how to engage with it, the musical instrument becomes a primary locus of agency, permeating our actions and intentions – which can thus be regarded as “affected.” There is a stage in the acquisition of instrumental musical skills wherein that agency is manifested, allowing the “patient” to shift to acting as an “agent” (Gell, 1998, p. 22).

The acquisition of instrumental musical skills is accompanied by the process of oscillation between affecting and being affected – “action and passion” – e.g., motor command based on auditory and tactile information. Through this oscillation, we develop resistance to the instrument’s materiality, and at the same time we establish direct tangible contact with the musical world that is created and shaped by this embodied musical activity. In this sense, such embodied musical activity can be conceived of as joined with the musical instrument, and the musical sounds being generated and shaped dynamically. In turn, the embodied activity gives rise to the pre-reflective bodily sense, which is accompanied by a double feedback loop consisting of auditory and kinesthetic/tactile feedback (Leman, 2007; Kim, 2010). For instance, we become used to the vibrotactile feedback of the musical instrument’s resonator through direct bodily contact or proximity; as a result, we attain bodily feelings while playing a musical instrument. We also learn instrumental musical skills using techniques that are appropriate to the controller (a keyboard, string, reed, etc.). Instrumental skills that are acquired are therefore closely related to the bodily techniques afforded and constrained by each musical instrument’s controller. A change in the controller’s characteristics – such as the material a string is made of, or resistance pressing a key – gives rise to an adjustment or new strategy of bodily techniques relying on our bodily sense, which, in routine embodied activity, remains pre-reflective.

This kind of pre-reflective bodily sense becomes aware if, and only if, our attention is directed toward the proximal, to apply Michael Polanyi’s term (Polanyi, 1966) – for instance, the bodily state while using bodily movements dependent on the various physical characteristics of the controller of the musical instrument, and/or a specific characteristic of the musical sounds generated and controlled by that instrument. While learning to play a musical instrument, we generally start in a state in which the proximal becomes the foreground; through the acquisition of instrumental musical skills, we eventually attain a state in which the proximal recedes into the background. This allows us to direct our attention to the distal – those qualities of musical sounds and the melodic and rhythmic structure of music shaped by bodily activity – in shaping musical sounds in a refined way and combining sound sequences with one another.

In his seminal work *The Tacit Dimension* (1966), Polanyi discusses the proximal and the distal, respectively, as the phenomenal and functional structure of what he refers to as “tacit knowing”; he coined this term to describe the ways in which we are subsidiarily aware of cranial processes (cf. Polanyi, 1966, p. xix), “as if they were part of our body” (Polanyi, 1966, p. xviii). His main claim is that cranial processes related to cognitive actions become meaningful if the transposition of spontaneously indwelling bodily experience (the proximal) into the perception of things outside (the distal) takes place (cf. Polanyi, 1966, p. 14 f.). How the proximal and the distal are combined to capture the meaningfulness of embodied musical activity involved in the acquisition of instrumental skills will be discussed in the following section, taking up concepts of body image and body schema recently discussed in the context of cognitive (neuro)science and phenomenology.

## Oscillations Between Body Image and Body Schema in the Acquisition of Instrumental Musical Skills

When learning to play a new musical instrument, we try to generate and control a musical sound that has a specific characteristic according to each instrument – such as timbre and vibrato – and to present sound sequences and to combine sound sequences to one another. The goal of acquiring instrumental musical skills could therefore be considered as musically meaningful activity guided by a shift of attention from the proximal toward the distal. This activity could also be understood in terms of recent theories of body schema and body image.

The body schema that Merleau-Ponty discusses is a system of sensory-motor capacities underlying habitual bodily actions. The system is integrated into extraintentional operations carried out prior to or outside of intentional awareness of one’s own body. The latter occurs “in terms of monitoring or directing perceptual attention to limb position, movement, posture, pleasure, pain, kinaesthetic experience, and so on […]” (Gallagher and Zahavi, 2008, p. 146); through an intentional operation of consciousness that constitutes aspects of a body image (Krois, 2011), the body becomes the content of consciousness. By contrast, body-schematic processes are characterized as “sensorimotor functions” (Gallagher and Zahavi, 2008, p. 146), operating “below the level of self-referential intentionality” (Gallagher, 1995, p. 228) involving pre-reflective awareness of our bodily movements and postures, rather than objectifying body-awareness (Gallagher, 2005, p. 24f.; Gallagher and Zahavi, 2008, p. 146), conscious bodily self-perception (Krois, 2011, p. 258) or a conceptual model of the body (Gallagher, 2005, p. 32). As such, body schema and body image can be distinguished from one another. However, body-schematic processes are conceived of as a basis for an intentional operation of conscious experience; the manifold aspects of sense-perceptible appearances of one’s own body are only unified based on the body schema so as to result in a complex body image (cf. Krois, 2011, p. 258).

The status of the body schema and body image oscillates during certain bodily activities, such as those devoted to the acquisition of instrumental musical skills. The first stage of learning a new musical instrument involves intentional action, for which we have novel modes of engagement with the world. At this stage, our body schema – which, for habitual actions, would function without conscious representations – needs to be reoriented. While the musical instrument remains opaque, and thus requires our attention toward the proximal, i.e., perceptions and feelings of the body, the process of monitoring of our instrumental musical skills takes place, which renders the body objectified. In other words, the body image is constituted while engaging in non-habitual actions; although the bodily states that we are aware of cannot be verbalized explicitly in this stage, we have conscious experience of those states. In an advanced stage, where the necessary instrumental techniques have been mastered, we reach the status of habitual bodily action, and the musical instrument becomes transparent (Jaeger and Kim, 2008; Nijs et al., 2013; Schiavio and De Jaegher, 2017). As a result, body image recedes into the background and we have pre-reflective, non-objectifying body-awareness. At this stage, our attention is shifted from the proximal (our bodily states) toward the distal (for instance, to the melodic and harmonic structure of the music shaped through our embodied activity).

## Relations of Bodily Gestures to the Distal

In paying attention to the distal, we are not only concerned with the qualities of single sounds, but also with a unity of music – such as the melodic and rhythmic structure, in which sound sequences are merged by virtue of their relation to one another. In shaping such a melodic and rhythmic structure, we are preparing the next sound – in terms of physical energy, corporeal postures and gestures – directly related to the sound occurring in now-moments. These corporeal postures and gestures include both those used while closing the sound that has almost passed, and those that prepare for applying a further instrumental technique to generate and control the next sound – all until the last sound of a unity of music has been created and shaped (Kim et al., 2010). Such micro-gestures used in the context of musical performance can be empirically investigated, most effectively by a high-speed camera or a motion-capture device (ibid.).

A possible mechanism underlying such musical structuring related to the now-moments might be what Mark Reybrouck characterizes as “in-time music” processing (Reybrouck, 2017), which takes place within “the moment-to-moment history of successive acts of focal attention” (Reybrouck, 2017, p. 89). This seems compatible with the Husserlian concepts of the primary memory and primal impression (Husserl, 1991 [1966]) that allow for extension of presence in regards to what has passed in now-moments (retention) and what will follow in now-moments (protention). Moreover, a growing body of research on the bodily gestures used in musical performance shows that corporeal macro-gestures are also used in shaping a musical unity. Tying in with David McNeill’s research on language-accompanying gesture (McNeill, 1992), such gestures include those that McNeill refers to as “iconic gestures” sharing a musically meaningful aspect – e.g., gestures imitating melodic or dynamic contour – or “beat gestures” emphasizing selected musical elements, serving to organize sound sequences as musical structure rather than conveying information related to musical meaning. In particular, those gestures accompanying bodily activity involved in the generation and control of musical sounds often exhibit a close relation to the characteristics of the related perceptible musical sounds – both of singular sounds and sound sequences that are experienced as a unity (Godøy and Leman, 2009). This kind of shaping of a musical unity could involve what Husserl calls “the secondary memory” (Husserl, 1991 [1966]), which goes beyond the extended presence, and is related to intermediate-term rather than short-term memory and working memory.

Often, such gestures are used involuntarily in the context of musical performance when our attention is directed toward the distal. There are, however, cases in which corporeal gestures share characteristics with the musical sounds generated and controlled by a musical instrument. In traditional South Indian (Karnatak) music, such gestures are part of instrumental education, used to help players imagine the characteristics of sounds and the musical structure – especially the svaras (scale degrees of a raga) – to be shaped (Pearson, 2016). Likewise, a Korean musical tradition observes a similar practice, albeit the other way around: The first step in learning to play a musical instrument is learning to produce typical instrumental sounds and rhythmic structures by “singing” sounds, allowing for embodiment of essential musical characteristics such as duration, contour and tension/relaxation in terms of imitation. The acquisition of instrumental body techniques follows, using the voice to imitate instrumental sounds, provided that the instrumental techniques are mastered in relation to the qualities of musical sounds and structures of music. Techniques imitating the characteristics of musical sounds that can be generated and controlled by a musical instrument, through the help of another medium, are considered embodied techniques allowing for maintenance of attention to the distal.

## Understanding the Forms of Our Own Experiences

In an advanced stage of the acquisition of instrumental musical skills, in which the body image does not play a significant role, the large-scale structure of music is shaped in a very refined manner – both in terms of the qualities of the musical sounds and the structuring unities of musical experience. Often accompanied by the flow of bodily gestures, performing a musical piece allows the player immersion into the musical world that is created by embodied activity. According to the philosopher Susanne K. Langer, this world has a structural analogy to the world of human feeling:

“The tonal structure we call “music” bears a close logical similarity to the forms of human feeling – form of growth and attenuation, flowing and stowing, conflict and resolution, speed, arrest, terrific excitement, calm or subtle activation and dreamy lapses – not joy and sorrow perhaps, but the poignancy of either and both – the greatness and brevity and eternal passing of everything vitally felt” (Langer, 1953, p. 27).

Langer’s unusual concept, encompassing all aspects of human vitality, is akin to our pre-reflective feelings in the world, which the contemporary philosopher Matthew Ratcliffe refers to as “existential feelings.” Existential feelings are concerned with bodily feelings; according to Ratcliffe, however, they are not just feelings of internal bodily states, but rather “ways of finding ourselves in a world, existential backgrounds that shape all our experiences” (Ratcliffe, 2008, p. 47). The musical structure exhibits forms of vitality (Stern, 2010), dynamic forms of a sentient being’s behavior in relation to others. Although music is not a sentient being, forms of vitality are assigned to a musical expressive Gestalt (Stern, 2010; Kim, 2013), which is considered to both result from the intra-musical relation (combination of musical elements with one another), and be related to the world and other people.

Shaping the musical structure, with the involvement of embodied activity, can make our pre-reflective feelings in the world reflective, in such a way that the forms of vitality unfolding in the world of human feeling are co-shaped while shaping the forms of vitality situated in the musical world. This state of shaping and co-shaping forms of vitality (Kim, 2013) through musical embodied activity is achieved in an advanced stage of learning how to play a musical instrument. The shift of our attention of the proximal to the distal while playing a musical instrument therefore does not simply mean that we attend to things external from things internal. Much more than that, it allows us to direct our attention to our being-in-the-world, dissolving a dichotomy between inner and outer.

## Conclusion and Discussion

In this paper, the bodily activity involved in the acquisition of instrumental skills was regarded not only as the process of learning *instrumental* skills related to a given musical instrument, but also that of learning *musical* skills and experiencing music itself. In line with phenomenological theories of embodied perception and cognition, the body was conceptualized neither as thing nor construct (List and Fiala, 1997; Barkhaus and Fleig, 2002), but as embodied being (Seifert and Kim, 2012). The initial thesis held that the body could also have a status of patient – according to Gell’s theory of agency – who suffers from the material resistance of a musical instrument relying on its materiality, whether physical (in the case of mechanical musical instruments) or virtual (in the case of digital musical instruments).

The extent to which the embodied musical activity that is directed toward learning instrumental musical skills, accompanied by the chiasmus of action and passion, was discussed, making clear that the process of oscillation between body image and body schema allows humans to acquire instrumental musical skills such an embodied way that they can attend from their bodily states – the proximal – to the qualities of musical sounds and the melodic and rhythmic structure of music that are shaped by their bodily activity – the distal. Such an embodied knowledge of those skills was considered manifest in bodily gestures, which prove to be both instrumental and related to musical properties – e.g., musical contour and tension/relaxation – in a non-causal and meaningful way. In turn, the extent to which the forms of one’s own experience are understood while the musical world is created by one’s bodily activity was examined. Tying in with Langer’s theory of music as a form of human feeling and Ratcliffe’s concept of existential feelings, it was argued that the characteristics of musical sounds that are experienced – as related to bodily gestures accompanying embodied activity involved in the generation and control of musical sounds – allow us to make our pre-reflective feelings in the world – “existential feelings” – reflective. This claim is in line with Langer’s theory of music as a form of human feeling and Stern’s concept of forms of vitality; these make clear that the pre-reflective forms of feelings in the world have a structural analogy to the qualities of musical sounds and the melodic and rhythmic structure of music that are shaped in a relational interaction with the world.

Playing a musical instrument can therefore be conceived of as an effective means to understand the forms of our own experience in relation to the musical world: it serves as musical worldmaking. Moreover, as recent theories of embodied music cognition of music support the thesis that covert gestures (“inner gestures”) and/or overt gestures (such as dancing) accompany the process of musical listening (Leman, 2007; Cox, 2016), it can be claimed that music perception, during musical listening, aids in understanding the forms of our own experience as related to the world. In other words, music perception can be characterized as a process of co-shaping the forms of human feeling or forms of vitality embodied in music (Stern, 2010; Kim, 2013), and thus of providing access to our existential feelings. The acquisition of instrumental musical skills that allows us to shape musical sounds and structure music, in tandem with our bodily (partially very refined) activity, could therefore enhance not only our sensorimotor capabilities, but also our capacity for relating our own experience to the world and others.

## Author Contributions

JK developed the main idea and wrote the manuscript.

## Conflict of Interest

The authors declare that the research was conducted in the absence of any commercial or financial relationships that could be construed as a potential conflict of interest.
